# Heterogeneous Solid-State Plasticity of a Multi-Functional Metallo-Supramolecular Shape-Memory Polymer towards Arbitrary Shape Programming

**DOI:** 10.3390/polym14081598

**Published:** 2022-04-14

**Authors:** Guancong Chen, Di Chen

**Affiliations:** 1 Ningbo Research Institute of Zhejiang University, Ningbo 315100, China; cc_chen@zju.edu.cn; 2 State Key Laboratory of Chemical Engineering, College of Chemical and Biological Engineering, Zhejiang University, Hangzhou 310027, China

**Keywords:** shape-memory polymers, metallo-supramolecular interactions, shape programming, self-welding, reprocessing

## Abstract

Shape-memory polymers (SMPs) exhibit notable shape-shifting behaviors under environmental stimulations. In a specific shape-memory cycle, the material can be temporarily fixed at diverse geometries while recovering to the same permanent shape driven by the elastic network, which somewhat limits the versatility of SMPs. Via dynamic metallo-supramolecular interactions, herein, we report a multi-functional shape-memory polymer with tunable permanent shapes. The network is constructed by the metallic coordination of a four-armed polycaprolactone with a melting temperature of 54 °C. Owing to the thermo-induced stress relaxation through the bond exchange, the SMPs can be repeatedly programmed into different geometries in their solid state and show the self-welding feature. Via further welding of films crosslinked by different ions, it will present heterogeneous solid-state plasticity, and a more sophisticated shape can be created after the uniform thermal treatment. With elasticity and plasticity in the same network, the SMPs will display programmable shape-shifting behaviors. Additionally, the used material can be recast into a new film which retains the thermo-induced plasticity. Overall, we establish a novel strategy to manipulate the permanent shapes of SMPs through solid-state plasticity and develop a multi-functional shape-shifting material that has many practical applications.

## 1. Introduction

Shape shifting is crucial for the survival of creatures such as the mimosa or Venus Flytrap during environmental changes [1]. Inspired by such biological intelligence, the past years have witnessed tremendous developments in synthetic shape-shifting materials, which now play essential roles in diverse fields, including aerospace [2,3,4], biomedical devices [5,6] and soft electronics [7,8]. Among them, shape-memory polymers (SMPs) show unique shape-changing behaviors from temporary shapes to permanent shapes, triggered by environmental stimulations, such as heat, light or magnetic fields [9,10,11]. Despite significant improvements in developing triple [12] and multiple shape-memory effects [13], which can achieve programming of two or more temporary shapes for the polymer, all will generally recover to the same permanent shape initially defined by the mold. Post switching the original shapes of SMPs can further promote the versatility of shape-shifting behaviors and innovate much more practical applications. However, owing to elastic networks, the manipulation of the permanent shape is quite challenging. 

Dynamic polymer networks show special stress relaxation behaviors through bond exchange stimulated generally by heat or light, which endow the elastic polymers with solid-state plasticity [14,15,16]. Such unique features have been applied to the materials towards self-healing [17,18,19], reprocessing [20,21] and micro-manufacturing [22,23,24]. Via introducing dynamic bonds, shape-memory networks can be post fabricated into different shapes and permanently fixed after stress relaxation. In this manner, dynamic disulfide SMP has been developed, and it exhibited UV-induced shape-fixing properties [25]. Forgoing the capability of the light permeation, thermo-responsive polycaprolactone and polyurethane shape-memory networks are also established, respectively, and show more sophisticated shape-shifting behaviors through origami and kirigami [26,27]. 

Furthermore, dynamic metal–ligand interactions are also applied to the SMPs. The high bonding energy ensures stability at room temperature, and it will present dynamic features after triggering by heat [28,29,30]. The carboxylic and amino groups are usually used to construct the metallo-supramolecular networks [31,32]. Recently, the polymer contained in the pyridine group has been developed and crosslinked by Zn^2+^ ions. The obtained film shows light-induced welding through photo-thermal conversions [33]. Furthermore, metallo-SMPs are also formed and show different shape-shifting behaviors together with self-welding features [34]. Under these circumstances, the permanent shapes can be manipulated by the metal–ligand bond exchange. In addition, stress relaxation kinetics can be easily adjusted by simply replacing the metal ions compared with conventional dynamic covalent bonds [35]. By controlling the diffusion of metal ions in a shape-memory network, the gradient solid-state plasticity has been achieved, and this led to a distinctive shape-shifting behavior [36]. However, the types of permanent shapes are still limited to simple geometries, including folding and twisting shapes. Although the gradient solid-state plasticity can extend the capability of shape manipulations, it has low efficiency and is restricted by ion diffusion kinetics. Therefore, a more adaptive strategy to arbitrarily program the permanent shapes and acquire different shape-shifting behaviors is still required. Here, we integrate the merits of physical metal–ligand interactions (e.g., self-welding, recasting) to reconstruct the permanent shapes of SMPs and establish the heterogeneous solid-state plasticity by welding different ion-doped materials, which can acquire more complex permanent shapes forgoing the ion diffusion. Combining the plasticity and elasticity, the SMPs will show distinct shape-shifting behaviors.

## 2. Materials and Methods

### 2.1. Materials

Chloro-2,2′:6′,2″-Tripyridine was acquired from Aladdin (Shanghai, China). 1,5,7-triazabicyclo [4.4.0] dec-5-ene, 4′- 1,3-propanediol, triethylamine, Ɛ-caprolactone, and acryloyl chloride were purchased from TCI chemicals (Tianjin, China). Pentaerythritol tetra(3-mercaptopropionate) (PTME) was purchased from Sigma-Aldrich (Shanghai, China). Unless otherwise stated, all chemicals were used as received.

### 2.2. Methods

#### 2.2.1. Synthesis of 4′-[2-(3-Hydroxyl) Propoxy]-2,2′:6′,2″-Terpyridine

4′-chloro-2,2′:6′,2″-terpyridine (2 g), 1,3-propanediol (2.9 g), KOH (2 g), and anhydrous dimethyl sulfoxide (20 mL) were added into a 50 mL flask and reacted under magnetic stirring at 70 °C for 48 h. The produced liquid was poured into deionized water (50 mL) for precipitation. Then, the pH was adjusted to 6.7 by gradually adding hydrochloric solution (10%). The liquid was totally removed by centrifugation, and the remaining solid was vacuum-dried at 70 °C for 48 h. The obtained product was dissolved in methanol at 70 °C and recrystallized at around 0 °C. The crystalline solid was obtained by centrifugation and vacuum-dried at 70 °C for 24 h. In order to acquire pour product, the above recrystallization-based purification process was repeated twice. The used molecule and the acquired product were characterized by ^1^H-NMR shown in Figure S3.

#### 2.2.2. Synthesis of Polycaprolactone with a Terpyridine end Group (Mn = 5000) 

The acquired hydroxyl-terminated terpyridine (1 g), Ɛ -caprolactone (17.2 g), and 1,5,7-triazabicyclo [4.4.0] dec-5-ene (0.5 wt%) were added into a 50 mL flask. The reaction was conducted at 120 °C in a nitrogen atmosphere for 10 h. The resulting polymer was dissolved in toluene and precipitated in cold methanol. The obtained product was vacuum-dried overnight at room temperature. The product was characterized by ^1^H-NMR shown in Figure S4.

#### 2.2.3. Synthesis of TPyA

The previously obtained polycaprolactone with a terpyridine end group (20 g), acryloyl chloride (1.08 g), and triethylamine (1.22 g) were co-dissolved in 100 mL toluene at 80 °C and reacted for 20 h. Following being precipitated in cold ethanol, the obtained solid product was vacuum-dried for 24 h at room temperature. The TPyA was tested by ^1^H-NMR shown in Figure S4.

#### 2.2.4. Synthesis of TPy-Ni^2+^ Networks

TPyA (2 g) and PTME (0.0489 g) were dissolved in N, N-dimethylformamide (DMF, 2 g). Triethylamine (2 wt%) was added subsequently. The mixture was reacted at 80 °C for 10 h. The acquired four-armed macro-monomer was characterized by ^1^H-NMR and ^13^C-NMR shown in Figure S1 and Figure S2, respectively. Then NiCl_2_·6H_2_O (0.0474 g) and DMF (2 g) were added into the liquid. The solution was stirred at 140 °C for 3 min, then transferred into a mold. On removing the solvent at 70 °C after 10 h, the dynamic film was acquired. By simply changing the mixing metal ions, different films could be obtained.

### 2.3. Characterization

The shape-memory property was characterized by a dynamic mechanical analyzer (DMA, type: TA Q800). The maximal force was 18 N and the resolution was 0.1 mN. Using stretching mode, the strain and stress could be auto detected. In addition, the temperature could be controlled by a temperature sensor. In order to avoid sliding, the sample was clamped tightly. Differential scanning calorimetry (DSC) measurements were conducted using a TA Q2000 machine under N_2_ at a temperature ramping rate of 5 °C/min. Mechanical tests were performed using a Zwick/Roell tensile machine at a stretching speed of 10 mm/min and the sample geometry was 25 mm × 5 mm × 0.3 mm. The applied force sensor was 1.5 kN, and it utilized physical clamps. 

## 3. Results and Discussion

**Figure 1:**
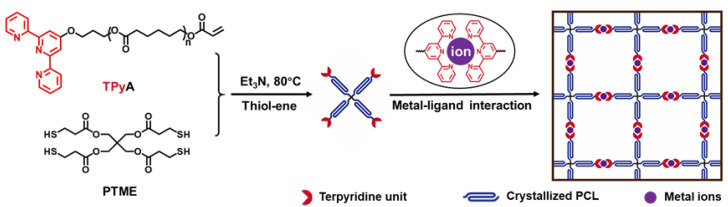
Process of constructing the metallo-supramolecular shape-memory network.

Here, we prepare a metallo-supramolecular shape-memory network, and establish diverse shape manipulation approaches using self-welding and solid-state plasticity. Specifically, the shape-memory polymer network is formed by the two-step methods shown in Figure 1. The initial four-armed macro-monomer was prepared through a thiolene click reaction of a polycaprolactone acrylate containing terpyridine (TPy) and pentaerythritol tetrakis (3-mercaptopropionate). After mixing with the metal ion solution, it turns to a shape-memory network via the metal–ligand interactions.

**Figure 2:**
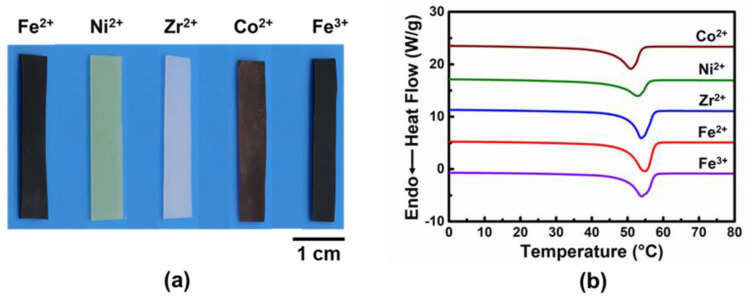
Features of acquired SMPs. (**a**) Appearance of the films doped with different metal ions. (**b**) The differential scanning calorimetry curves.

TPy can coordinate with diverse metal ions, and the colors of the obtained films are changed accordingly, as can be seen in Figure 2a. On the contrary, the transition temperatures are all around 54 °C, which was derived from the melting of crystalized polycaprolactone (PCL) domains (Figure 2b). Since the utilized polycaprolactone is at the same molecular weight, the relative transition temperatures are similar.

**Figure 3:**
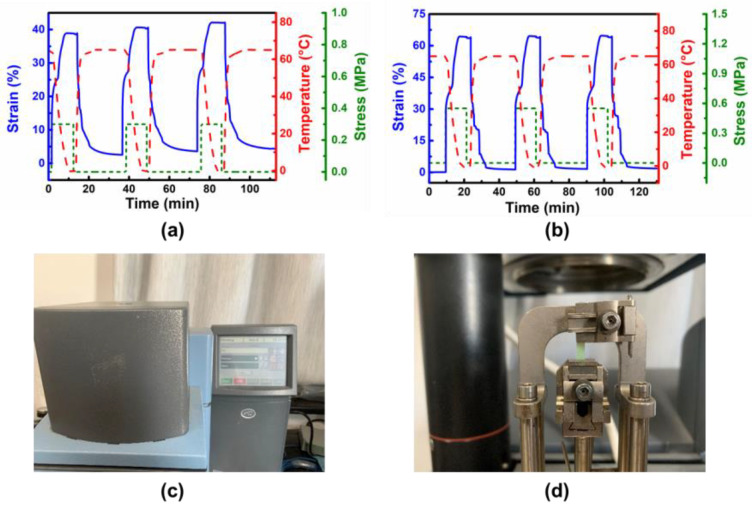
Shape-memory properties of samples doped by Ni^2+^ (**a**) and Co^2+^ (**b**). The photos of the DMA machine (**c**) and the loading cabin (**d**).

Generally, the phase transition is the key issue for the shape-memory effect. Figure 3a indicates three typical shape-memory cycles of the film doped with Ni^2+^. Specifically, the Ni^2+^-dopped sample was heated to 65 °C for melting PCL. In the meantime, the sample was stretched under 0.3 MPa stress. After cooling to 0 °C, the sample was fixed at a strain of 38.8%. Following removal of the applied stress, the sample recovered slightly, and the final strain was fixed at 38.6%. Thus, the fixing ratio is 99.5%. When re-heating to 65 °C, the sample recovered to its original state because of the melting of PCL, and the recovery ratio was 93%. The same process was applied to the Co^2+^ doped sample, which showed decent shape-memory behavior with the fixing ratio at 99.6% and a recovery ratio at 98%, as shown in Figure 3b. Figure 3c,d show the testing machine and loading cabin.

**Figure 4:**
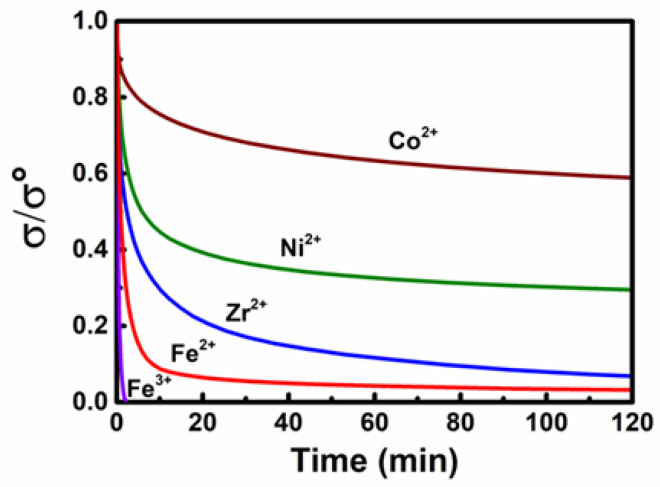
The stress relaxation kinetics of different metal ions doped samples at 60 °C. σ represents the instantaneous stress and σ° represents the initial stress.

Owing to the dynamic nature of metal–ligand interactions, the SMPs can undergo a thermo-induced bond exchange, which can release internal stress, thus realizing the solid-state plasticity. Meanwhile, the previous stress relaxation kinetics is determined by the utilized metal ions. As seen in Figure 4, at 60 °C, which is close to the melting temperature, samples doped with Fe^3+^, Fe^2+^ and Zr^2+^ can rapidly relax the stretched networks. Under these circumstances, there is a significant adverse impact on the shape-memory behaviors, which rely on the elasticity of the networks. On the contrary, the stress relaxation rates of the film containing Co^2+^ are too slow. By comparison, the polymer network doped by Ni^2+^ possesses an acceptable relaxation rate, and exhibits distinctive shape-memory behaviors as clearly shown in Figure 3a above. Therefore, due to the controllable elasticity and plasticity of Ni^2+^ doped films, we generally used them as the model materials for the following demonstrations.

**Figure 5:**
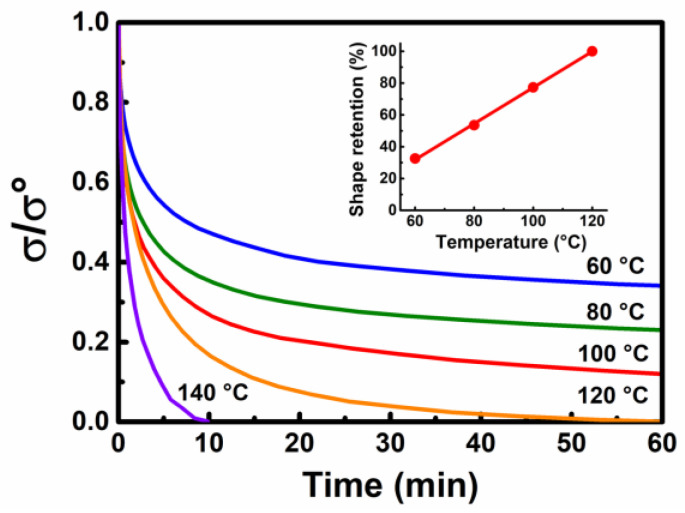
The stress relaxation properties of Ni^2+^-doped films at different temperatures, and inserts show the shape retention.

Solid-state plasticity is further influenced by temperatures. As shown in Figure 5, the relaxation rates of Ni^2+^-doped samples accelerated along with the increase in temperature. In addition, the shape retention increased from 30% at 60 °C to 100% at 120 °C. Consequently, we can set the network into an adaptive capability of plasticity by switching temperatures in a specific shape-shifting process. 

**Figure 6:**
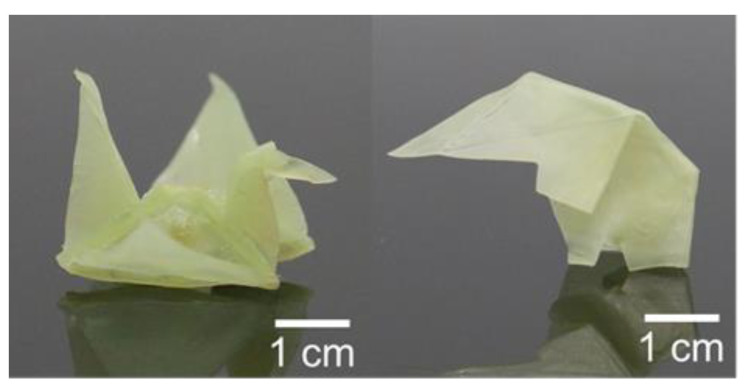
SMPs with permanent shapes of a bird and an elephant, respectively.

Using the up-mentioned solid-state plasticity, we can program the permanent shapes of a shape-memory polymer. Utilizing the Ni^2+^-doped samples, the 2D film can be folded into different 3D geometries and permanently fixed by heating at 140 °C for 10 min. Using this method, bird- and elephant-shaped SMPs can be obtained (shown in Figure 6).

**Figure 7:**
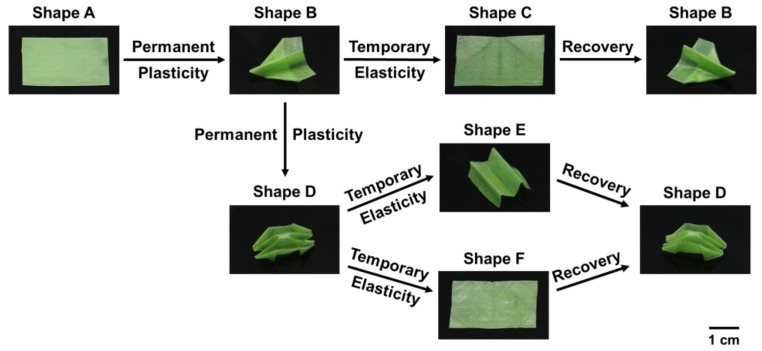
The diverse shape-shifting behaviors via a metallo-supramolecular shape-memory film.

With distinctive elasticity and plasticity in our metallo-supramolecular shape-memory networks, they can exhibit unique shape-shifting behaviors in a more programmable way. As seen in Figure 7, a rectangular film (Shape A) is folded and permanently fixed into an airplane shape (Shape B) by heating it at 140 °C for 10 min. When the temperature switches to 65 °C, it turns to its rubbery state because of the melting of the PCL domain and can be opened to a planar film (Shape C), which is temporarily locked by cooling. After re-heating to 65 °C, the planar film will recover to the airplane shape (Shape B) again due to its elasticity. In addition, the polymer with an airplane shape (Shape B) can be reprogrammed into a boat shape (Shape D) via solid-state plasticity under 140 °C and can be temporarily changed to either a curved structure (Shape E) or a planar structure (Shape F) by crystallization of PCL domains, which will all recover to the boat shape over the melting temperature. The above diverse shape-shifting behaviors exactly present the merits of our metallo-supramolecular networks. The shape memory process can be triggered at a relatively low temperature, while the permanent shape can be programmed at a higher temperature. Both can be independently regulated. Theoretically, the number of obtained shape-shifting modes is infinite.

**Figure 8:**
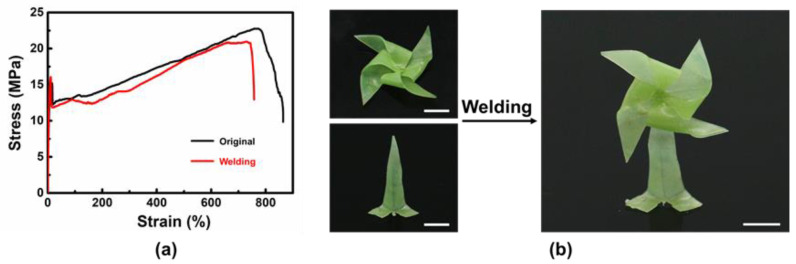
The self-welding of metallo-supramolecular networks. (**a**) The mechanical properties of an original sample and a welded sample. (**b**) A windmill tower shape obtained from self-welding of different parts, scale bar: 1 cm.

Physical metallo-supramolecular interactions further provide the networks with self-welding properties that can create more complex forms via assembly. As indicated in Figure 8a, the mechanical properties of films before and after welding are very similar, revealing good self-welding capabilities. Thus, as shown in Figure 8b, we programmed the planar films into a windmill shape and a shaft shape, respectively, by solid-state plasticity. The two parts were further assembled into a windmill tower through thermo-induced self-welding. In this way, more sophisticated shapes can be established beyond conventional origami [37].

**Figure 9:**
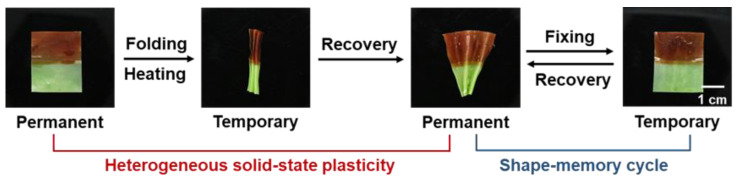
Shape-shifting of a welded film with two different parts.

Utilizing macroscopic welding of blocks with different stress relaxation kinetics, heterogeneous solid-state plasticity, can be realized, which extends the shape-memory behaviors. As shown in Figure 9, a Ni^2+^-doped film (light green) and a Co^2+^-doped film (dark brown) were welded and uniformly folded. After heating at 140 °C for 10 min, the bottom Ni^2+^-doped part was totally relaxed, while the top Co^2+^-doped part still possessed internal stress. Because of this imbalance, a permanent fan shape was obtained. With subsequent heating and cooling, the film could be temporarily fixed at the planar sheet, which recovered to the fan shape over the melting temperature. Such shape-memory behavior can be repeatedly triggered because of the elasticity. As a result, it establishes the heterogeneous solid-state plasticity by film welding, which no longer needs to control ion diffusion to achieve the spatial manipulation of plasticity [31].

**Figure 10:**
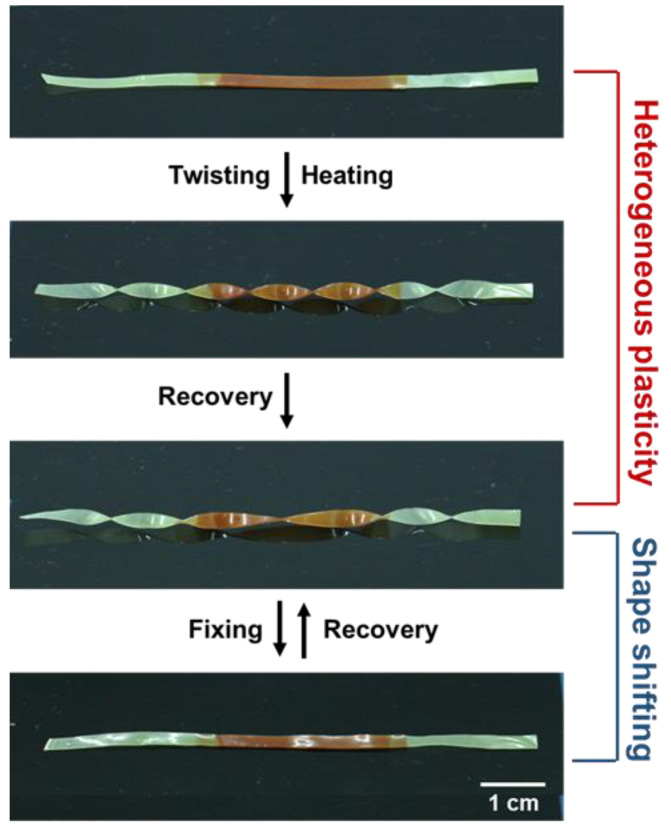
Shape manipulations of a sample with triple blocks.

In addition, a sample with triple blocks is also established in Figure 10. Via the homogeneous thermal treatment at 140 °C, two ends were totally fixed, while the central zone was only partially locked; thus, a new permanent shape was constructed. Meanwhile, the sample still maintains the shape-memory property because of the elastic network. The idea of welding film blocks crosslinked by different metal ions can construct complex, permanent shapes of SMPs and absolutely extend the versatility of shape-shifting behaviors.

**Figure 11:**
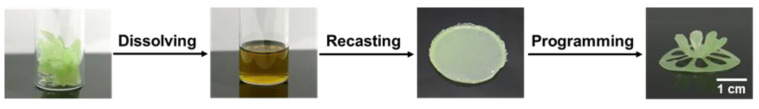
The recasting and reprogramming of the used sample.

Finally, the used materials can be dissolved in dimethylformamide at 150 °C and recast into a new film, owing to the dissociation of dynamic metal–ligand bonds during thermo-induced exchange. The process is shown in Figure 11. After recasting, the newly formed film maintains the capability of solid-state plasticity and can be permanently programmed into another shape. Generally, this allows the reuse of material resources.

## 4. Conclusions

In summary, we have prepared a novel metallo-supramolecular shape-memory polymer network crosslinked by metal–ligand bonds. Derived from the thermo-induced bond exchange, the network can be programmed into arbitrary permanent shapes in its solid state, which presents solid-state plasticity. Moreover, with plasticity and elasticity in the same network, it shows distinctive shape-shifting behaviors. With further welding of shape-memory polymer blocks holding different stress relaxation kinetics, a more complex form can be obtained via the heterogeneous solid-state plasticity after the uniform thermal treatment, which distinguishes this research from other currently reported shape programming approaches. Final reprocessing by dissolving and casting improves the capability of shape manipulations and increases the efficiency of resource utilization. By applying the established strategy on adaptive shape manipulations, we envision that our multi-functional dynamic SMPs can be applied to many deployable devices. In addition, the idea of assembling diverse blocks into a new film can achieve the spatial selectivity of different properties (such as modulus, wetting) after uniform thermal treatments.

## Data Availability

The data presented in this study are available in the article.

## Supplementary Materials

The following supporting information can be downloaded at: https://www.mdpi.com/article/10.3390/polym14081598/s1, Figure S1: The ^1^H-NMR spectrum of the four-armed macro-monomer; Figure S2: The ^13^C-NMR spectrum of the four-armed macro-monomer; Figure S3: The ^1^H-NMR spectrum of the Chloro-2,2′:6′,2″-Tripyridine (left) and hydroxyl terminated TPy (right); Figure S4: The ^1^H-NMR spectrum of the polycaprolactone with a terpyridine end group (left) and TPyA (right).
