# Supplementary material for: Heterogeneous Solid-State Plasticity of a Multi-Functional Metallo-Supramolecular Shape-Memory Polymer towards Arbitrary Shape Programming
Source: Polymers (Basel). 2022 Apr 14;14(8):1598. doi: 10.3390/polym14081598 (PMC9027464; doi:10.3390/polym14081598)
Supplement: Supplementary file 1 [file polymers-14-01598-s001.zip › polymers-1664731-supplementary.pdf]

Supporting Information

Heterogeneous solid-state plasticity of a multi-functional metallo-supramolecular shape-memory polymer towards arbitrarily shape programming

Guancong Chen[1,2], Di Chen[1,2,*]

[1] Ningbo Research Institute of Zhejiang University, Ningbo 315100, China; 11928058@zju.edu.cn (G. Chen)
[2] State Key Laboratory of Chemical Engineering, College of Chemical and Biological Engineering, Zhejiang University, Hangzhou 310027, China.

* Correspondence: di_chen@zju.edu.cn

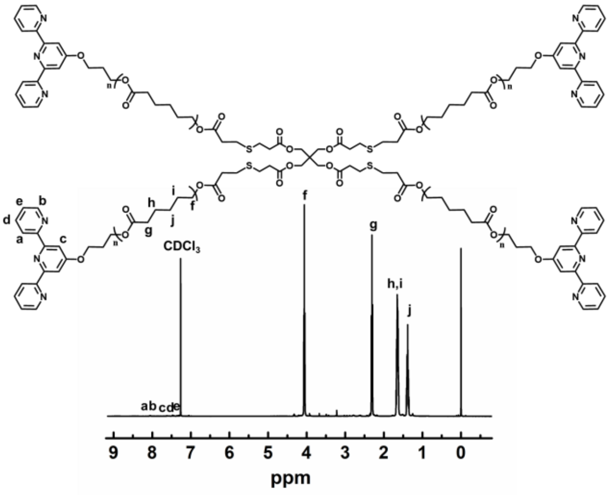

Figure S1. The ^{1}H-NMR spectrum of the four-armed macro-monomer.

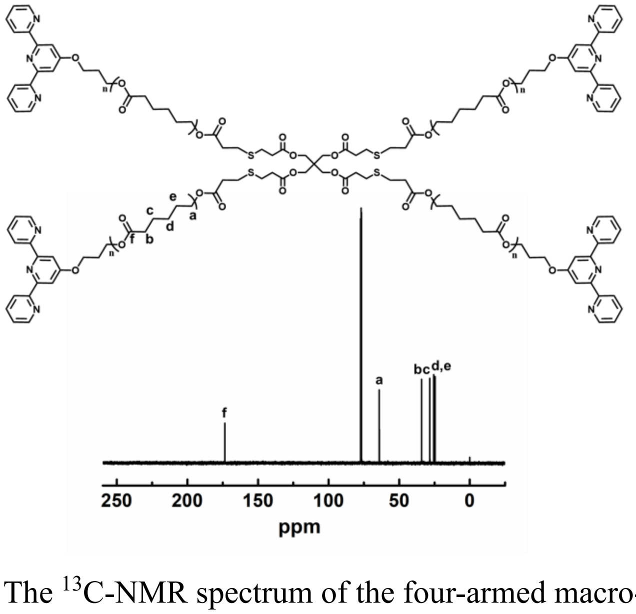

Figure S2. The ^{13}C-NMR spectrum of the four-armed macro-monomer.

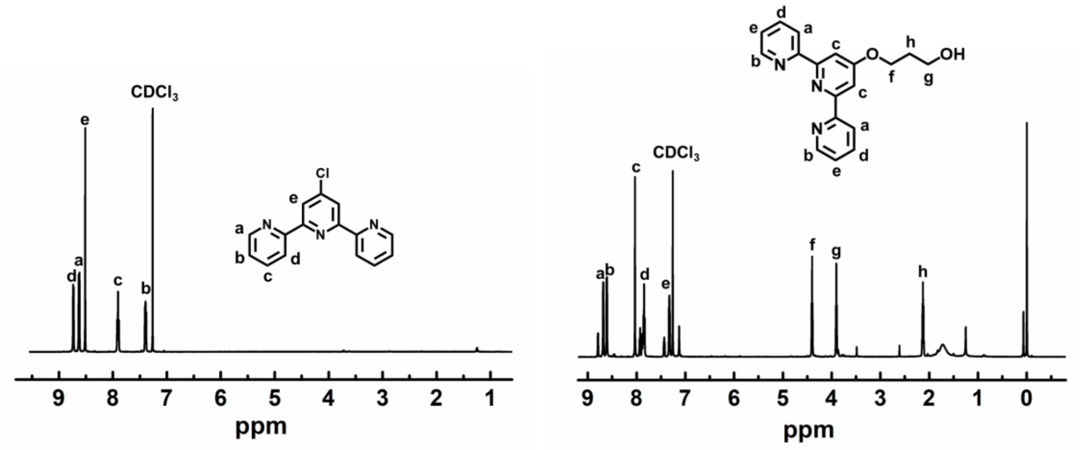

Figure S3. The ^{1}H-NMR spectrum of the Chloro-2,2':6',2"-Tripyridine (left) and hydroxyl terminated TPy (right).

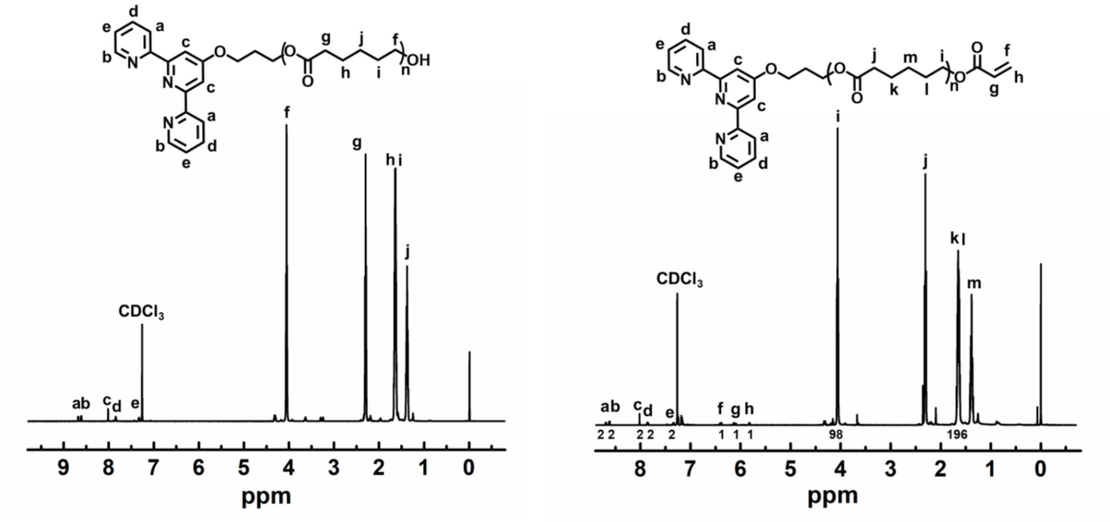

Figure S4. The ^{1}H-NMR spectrum of the polycaprolactone with a terpyridine end group (left) and TPyA (right).
